# Age-period-cohort projection of trends in blood pressure and body mass index in children and adolescents in Hong Kong

**DOI:** 10.1186/s12887-020-1928-2

**Published:** 2020-01-29

**Authors:** Man Ki Kwok, Irene Oi Ling Wong, C. Mary Schooling

**Affiliations:** 1 School of Public Health, Li Ka Shing Faculty of Medicine, The University of Hong Kong, 1/F, Patrick Manson Building (North Wing), 7 Sassoon Road, Hong Kong Special Administrative Region, China; 2 Graduate School of Public Health and Health Policy, City University of New York, New York, USA

**Keywords:** Child, Blood pressure, Body mass index, Trends, Age-period-cohort, Projection

## Abstract

**Background:**

Blood pressure (BP) and body mass index (BMI) trends during childhood and adolescence are complex, making context-specific projections necessary to inform prevention and presage changes.

**Objective:**

This study aimed to project BP and BMI in Hong Kong Chinese children and adolescents from 2015 to 2024 based on trends in BP and BMI observed from 1996/99 to 2014.

**Methods:**

We decomposed recent trends into sex-specific contributions of age, period and cohort using age-period-cohort linear regression with Bayesian inference and autoregressive priors based on BP in children and adolescents aged 9–18 years from 1999 to 2014 and BMI in those aged 6–18 years from 1996 to 2014. We then used the resultant models to project BP and BMI from 2015 to 2024.

**Results:**

During the study period, systolic BP decreased from 1999 to 2004/5 before gradually increasing to 2014 during childhood (for boys: from 104.6 to 101.9 and then to 103.4 mmHg) and during adolescence. Similar patterns were observed for diastolic BP. BMI generally increased from 1996 to 2009 before falling to 2014 during childhood (e.g. for boys: from 17.2 to 18.0 and then to 17.1 kg/m^2^). From 2015 onwards, systolic BP was projected to increase in girls, but remain stable in boys. For both sexes, diastolic BP was projected to increase, whereas BMI was projected to decrease to 2024.

**Conclusions:**

In this economically developed Chinese setting, future trends in BP and BMI in children and adolescents are predicted to be divergent, consistent with prior discordant trends in BP and BMI.

## Background

Projections of trends in blood pressure (BP) and body mass index (BMI) in children and adolescents may presage any future cardiovascular disease epidemic [1]. Discordant trends in BP and BMI have commonly been observed in long-term economically developed settings. In the United States, BP fell whilst BMI continuously rose in children and adolescents from 1963 to 1988 [2]. These predate similar trends in adults where systolic BP decreased whilst BMI increased from 1980 to 2008 [3]. Ischemic heart disease and ischemic stroke were relatively more common than haemorrhagic stroke in developed settings from 1990 to 2013 [4]. Given BMI is associated with higher risk of ischemic stroke but lower risk of hemorrhagic stroke [5] and uncontrolled BP is a major risk factor for hemorrhagic stroke [6], the divergent trends in BP and BMI in children and adolescents could be a sentinel for the future cardiovascular disease burdens.

Such divergent trends may indicate different drivers of BP and BMI, and contrasts between Western and non-Western settings may provide important insights about cardiovascular diseases. Unlike in the West, in Asia ischemic heart disease and ischemic stroke are relatively less common, but haemorrhagic stroke is more prevalent despite a relatively non-obese populations [4, 7]. However, whether BP and BMI trends may presage the cardiovascular disease burden in Chinese settings is under-studied. Concurrent rising trends of BP and BMI in children and adolescents were observed in Mainland China in the 1990s and 2000s [8, 9]. With China’s rapid economic growth over the last two decades, the previous trends may be difficult to relate to future trends. No study has examined potential drivers of BP and BMI trends in children and adolescents using an age-period-cohort (APC) analysis, which allows identification of the relative contribution of contemporaneous population-wide factors and cohort-specific exposures to the trends so as to inform interventions.

Hong Kong acts as an important sentinel in presaging cardiovascular health of a significant proportion of the global population living in the rest of China undergoing rapid economic growth. Hong Kong children and adolescents are the first generation to grow up in a developed Chinese city, with living standards and social infrastructure similar to Western Europe [10]. But their parents and grandparents experienced the transition from pre- to post-industrial living conditions in a lifetime [11]. Projections of BP and BMI trends in Hong Kong children and adolescents may help anticipate prevalence of associated diseases in future and inform allocation of health care resources. Moreover, decomposing the recent trends into the relative contribution of contemporaneous population-wide factors and cohort-specific exposures using APC analysis may help formulate more targeted public health initiatives and more effective physicians’ health advice, considering conventional adult lifestyle and medical care cannot explain the existing cardiovascular mortality trends [12]. This study primarily aims to use APC analysis to generate projections of BP and BMI trends and secondarily to decompose the recent changes in BP and BMI into sex-specific effects of age, period and birth cohort using population-representative BP in children and adolescents aged 9–18 years from 1999 to 2014 and BMI in those aged 6–18 years from 1996 to 2014 in Hong Kong, China.

## Methods

### Data source

This study used routinely collected BP and BMI from the Student Health Service of the Department of Health, which provides free annual health assessments for school-aged children and adolescents in Hong Kong [13]. The inclusion criterion was all primary and secondary day school students. The exclusion criterion was institutionalized children and adolescents with serious conditions requiring long-term hospitalization and not attending school. In Hong Kong, 9 years free universal public education (primary and 3-year junior secondary) has been provided since 1978 and 12 years (plus 3-year senior secondary) since 2008/09 [14]. The Student Health Service was introduced in 1995/96 for primary school students, and was extended to secondary school students in 1996/97, but was suspended for secondary school students in year 2 and above in 2009/10 because of the Human Swine Influenza Vaccination Programme. Students are encouraged to attend the health assessments voluntarily. Systematic difference in attendance by family socioeconomic position or related attributes is unlikely because the Student Health Service is free and enrolls students from all public and private schools in Hong Kong. The participation rate from 1995/96 to 2013/14 was 83.4% [15]. The health assessments include bi-annual assessments of BP (Primary 5 (age 10–11 years) onwards) and annual measurements of weight and height (Primary 1 (age 6–7 years) onwards). A single BP measurement was taken by nurses on the right arm in a seated position after more than 10 min of rest following a standard protocol with an age and size appropriate cuff size using an automated oscillometric device. Initial systolic or diastolic BP higher than the 90th percentile for sex, age and height based on local references was re-checked by physicians with a sphygmomanometer after 15 min of rest and this second measurement was recorded. Considering the Student Health Service serves a very large number of primary and secondary school students in Hong Kong, in order to facilitate close monitoring and service referral during the annual health assessments, no repeated measurement using the same instrument after a time period were performed. Nonetheless, the same protocol was used during the study period and hence would not affect the comparisons of BP across time. Height without shoes was measured by stadiometer to the nearest 0.1 cm and weight without shoes and outer clothing was measured by digital scales to the nearest 0.1 kg. BMI was calculated as weight in kilogram divided by height in meters squared. Coverage was incomplete in the early years, so we considered trends of BP since 1999 and BMI since 1996. We randomly selected one time point per participant so that there is no correlation between multiple measurements for the same participant. Given BMI was measured more often than BP across a wider time period and age range, more children and adolescents with BMI than BP were included. The large sample size for BP and BMI allows precise mean estimation for each sex- and age-period-cohort specific stratum and hence facilitates trend comparisons.

### Age-period-cohort

We considered age in years to reflect rapid physical growth and pubertal development during childhood and adolescence, and each examination year to reflect changes in living conditions and public health initiatives. This resulted in overlapped birth years because children or adolescents at the same age attending health check-ups in the same school year can be born in adjacent birth years e.g. a child aged 10 years attending a check-up in 2014/5 could be born in 2004 or 2005 and was categorized as birth year “2004–2005” (labeled as “2004” on the graphs). For BP, we had 10 ages from 9 to 18 years, 16 examination years from 1999 to 2014, and 25 cohorts born from 1980 to 2005. For BMI, we had 13 ages from 6 to 18 years, 19 examination years from 1996 to 2014, and 31 cohorts born from 1977 to 2008.

### Sample size calculation

For BP with 160 age-period-cohort specific strata, there were 196,299 boys and 205,741 girls in total i.e., on average 1226 boys and 1285 girls were available in each stratum. Given there were 51 parameters (10 age + 16 periods + 25 cohorts) in the age-period-cohort linear regression models, the sample size allowed detection of a small effect size of 0.022, with 80% power at 5% alpha.

For BMI with 247 age-period-cohort specific strata, there were 957,577 boys and 941,239 girls in total i.e., on average 3876 boys and 3810 girls were available in each stratum. Given there were 63 parameters (13 ages + 19 periods + 31 cohorts) in the age-period-cohort linear regression models, the sample size allowed detection of a small effect size of 0.012, with 80% power at 5% alpha.

### Statistical analysis

Considering the developmental differences between boys and girls at puberty, the analyses were stratified by sex. To decompose the secular trends in BP from 1999 to 2014 and BMI from 1996 to 2014 into the effects of age, calendar period and birth cohort, we fitted sex-specific age-period-cohort linear regression models with Bayesian inference, from which second-order changes i.e., changes in slope or inflection points only are interpreted [16], and used the fitted model to project future trends in BP and BMI from 2015 to 2024. As previously [17, 18], we constrained the second and penultimate periods and the central birth cohort to be the reference categories with no constraints on age, so as to generate identifiable estimates for period and birth cohort. Bayesian inference uses prior probability and likelihood functions to derive the posterior distribution of model parameters. We assumed mean BP and BMI followed a normal distribution. We specified second-order Gaussian autoregressive non-informative uniform priors in the forward direction for the age, period and cohort effects [19]. These priors specifying the initial expected value of each effect was based on an extrapolation from its two immediate predecessors, and provided nonparametric smoothing of the estimated effects by making a priori belief in smoothness. We extrapolated 10 additional period and cohort effects so as to allow projections of future BP and BMI trends.

We estimated the model parameters with Bayesian inference using Markov Chain Monte Carlo simulations with 5 concurrent chains started at different initial values since comparison of multiple chains allows discerning convergence. We used criteria R-hat to monitor convergence [20]. Based on the R-hat value, we discarded the initial samples as a burn-in period, and then sampled from the posterior distributions of the parameter estimates. The fitted and projected parameter estimates were summarized as posterior means and 95% credible intervals. The model goodness-of-fit with different combinations of age, period and cohort effect was measured by the posterior mean deviance. To compare fitted models for projection, the deviance information criterion (DIC) was calculated, which adjusts the posterior mean deviance for the number of parameters in the model [21]. A smaller DIC implies a better fit. Further technical details are shown in Additional file 1. For visual inspection, we plotted the fitted age, period and cohort effects with projections and examined the inflection points. In addition, we plotted the observed and projected curvature components to clarify when second-order changes (inflection points) occur. We also plotted the observed and projected mean BP or BMI to examine the overall trends. We performed jointpoint regression analysis using modified Bayesian information criterion to identify the calendar year or age at which the slope of the overall trends or estimated APC effects on BP and BMI changed significantly [22].

Statistical analyses were performed using R version 3.0.1 (R Development Core Team, Vienna, Austria) and the jointpoint trend analysis version 4.2.0.1 (National Cancer Institute, USA) [23].

## Results

A total of 196,299 boys and 205,741 girls aged 9–18 years with BP measurements from 1999 to 2014 and 957,577 boys and 941,239 girls aged 6–18 years with BMI measurements from 1996 to 2014 were included for aggregating into 160 mean BP and 247 mean BMI for each age-period-cohort specific stratum separately for boys and girls. Table 1 shows the mean BP and BMI across years stratified by sex and age. Systolic BP decreased from 1999 to 2004 before gradually increasing to 2014 for boys (from 104.6 to 101.9 and then to 103.4 mmHg) and for girls (from 104.2 to 101.9 and then to 102.9 mmHg) during childhood (9–11 years). Systolic BP also decreased from 1999 to 2005 before gradually increasing to 2014 for boys (from 115.0 to 113.3 and then to 114.9 mmHg) and for girls (from 108.9 to 106.6 and then to 108.1 mmHg) during adolescence (12–18 years). Similar patterns were found for diastolic BP. BMI generally increased from 1996 to 2009 before falling to 2014 for boys (from 17.2 to 18.0 and then to 17.1 kg/m^2^) and for girls (from 16.7 to 17.1 and then to 16.3 kg/m^2^) during childhood. BMI generally increased throughout during adolescence. Further, mean systolic BP was very similar for boys and girls during childhood (aged 9–11 years), whereas the sex difference in systolic BP gradually appeared during adolescence (aged 12–18 years), consistent with the blood pressure standards from the United States National High Blood Pressure Education Group in 2004 [24].

**Table 1:** Mean blood pressure (BP) and mean body mass index (BMI) across years stratified by sex and age using the Student Health Service (SHS) in Hong Kong

		Systolic BP					Diastolic BP					BMI			
		9–11 years		12–18 years			9–11 years		12–18 years			9–11 years		12–18 years	
Year	Participation rate at SHS	Boys	Girls	Boys	Girls		Boys	Girls	Boys	Girls		Boys	Girls	Boys	Girls
1996	86.3%											17.2	16.7	18.9	19.0
1997	74.7%											17.0	16.5	19.6	19.6
1998	77.9%											17.2	16.6	19.5	19.4
1999	76.0%	104.6	104.2	115.0	108.9		60.5	60.2	63.5	62.7		17.3	16.6	19.5	19.4
2000	75.6%	104.8	104.4	115.6	108.6		59.5	59.3	62.8	62.0		17.3	16.6	19.6	19.4
2001	76.8%	103.9	104.0	115.4	108.3		58.8	58.8	62.1	61.3		17.3	16.7	19.6	19.4
2002	79.0%	103.7	103.7	115.6	108.1		58.8	58.7	62.4	61.3		17.3	16.7	19.5	19.3
2003	80.2%	102.3	102.7	114.8	107.6		58.1	58.3	61.9	60.9		17.5	16.8	19.7	19.4
2004	81.5%	101.9	101.9	113.7	106.9		57.9	58.0	61.5	60.7		17.5	16.8	19.8	19.5
2005	78.6%	102.0	102.1	113.3	106.6		57.8	58.0	61.2	60.3		17.6	16.9	19.8	19.5
2006	83.6%	102.7	102.1	113.9	107.1		58.6	58.4	61.9	61.0		17.7	16.9	19.9	19.6
2007	87.0%	102.5	102.7	114.3	107.6		58.6	58.9	62.0	61.2		17.8	17.0	19.9	19.6
2008	87.5%	102.7	102.1	114.0	107.2		58.6	58.5	61.8	61.0		17.9	17.1	20.0	19.7
2009	87.1%	103.0	102.8	113.8	107.4		58.8	58.8	61.8	61.1		18.0	17.1	20.0	19.7
2010	95.1%	103.6	102.8	113.9	107.6		59.3	59.0	62.0	61.3		17.8	17.0	20.2	19.8
2011	89.0%	103.1	102.2	115.2	107.9		58.8	58.6	62.3	61.3		17.8	16.9	20.3	19.9
2012	88.3%	103.6	102.6	115.6	107.9		59.2	58.8	62.3	61.3		17.5	16.7	20.3	19.9
2013	90.0%	103.6	102.8	115.3	108.2		59.3	59.0	62.4	61.5		17.4	16.6	20.3	20.0
2014	90.8%	103.4	102.9	114.9	108.1		59.2	59.1	62.6	61.6		17.1	16.3	20.3	19.9

**Fig. 1:**
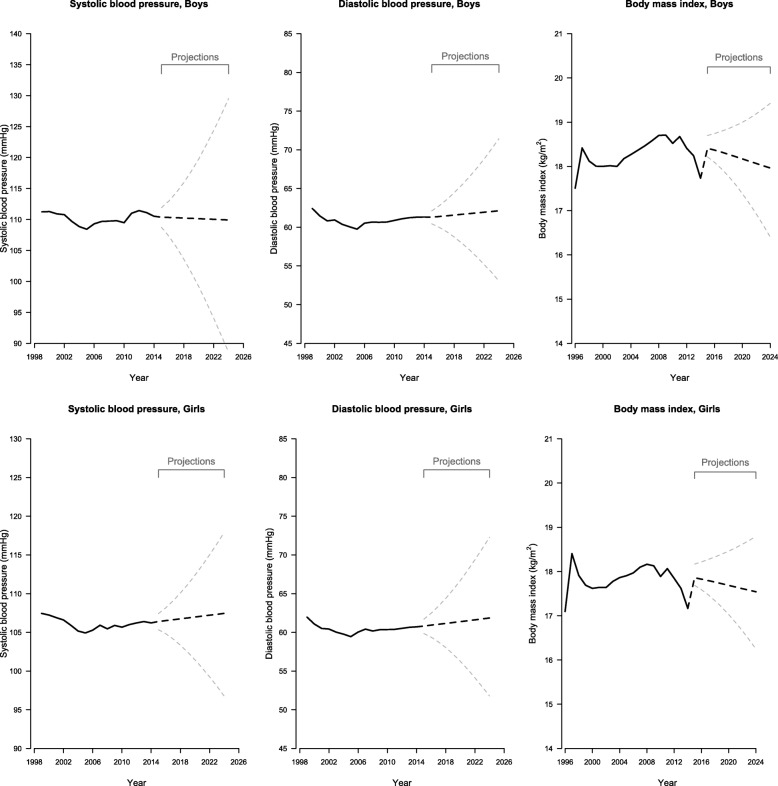
Systolic (Left) and Diastolic (Middle) Blood Pressure Among Boys (Top Panel) and Girls (Bottom Panel) Aged 9 to 18 From 1999 to 2014 and Body Mass Index (Right) Among Boys and Girls Aged 6 to 18 From 1996 to 2014 (Black Solid Lines) and Projected Blood Pressure and Body Mass Index (Black Dotted Lines) to 2024 With 95% Credible Intervals (Grey Dotted Lines) in Hong Kong Using Age-Period-Cohort Linear Regression with Bayesian Inference

Figure 1 shows the mean systolic and diastolic BP by year. Mean BP fell from 1999 to 2004/2005 and then gradually increased to 2014, except mean systolic BP in boys which started to fall again from 2012. For both sexes, mean BMI rose between 1996 and 1997, which reflected the gradual uptake of the Student Health Service first for children and then for adolescents during the same school year 1996/97. It then decreased before rising during 2000s and then falling to 2014.

**Fig. 2:**
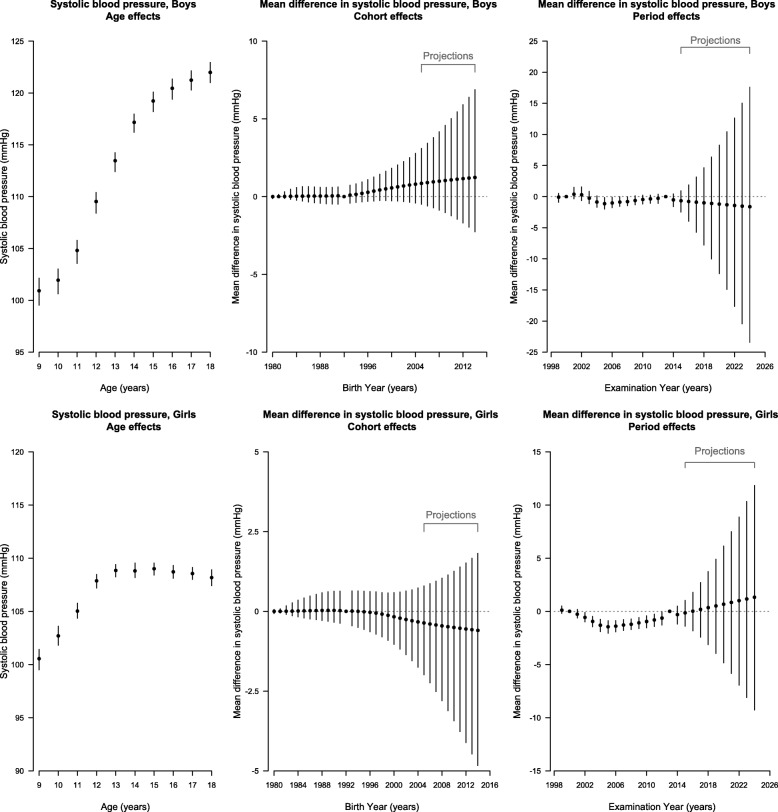
Parameter Estimates of Age, Period and Cohort Effects (Black Points) on Systolic Blood Pressure With 95% Credible Intervals (Vertical Lines) Among Boys (Top Panel) and Girls (Bottom Panel) in Hong Kong Using Age-Period-Cohort Linear Regression with Bayesian Inference. Left (Age Effects): Estimated Systolic Blood Pressure at Each Age From 9 to 18 Years. Middle (Cohort Effects) and Right (Period Effects): Estimated Mean Difference in Systolic Blood Pressure for Each Birth Cohort Born From 1980–1981 to 2004–2005 (Labeled as Earliest Birth Year for Each Cohort Group) With Projected Cohort Effects for Birth Cohort Born from 2005–2006 to 2014–2015 and for Examination Periods From 1999 to 2014 With Projected Period Effects from 2015 to 2024. The Second (2000) and the Penultimate (2013) Periods and the Central Birth Cohort (1992–1993) Were Specified as the Reference Categories

**Fig. 3:**
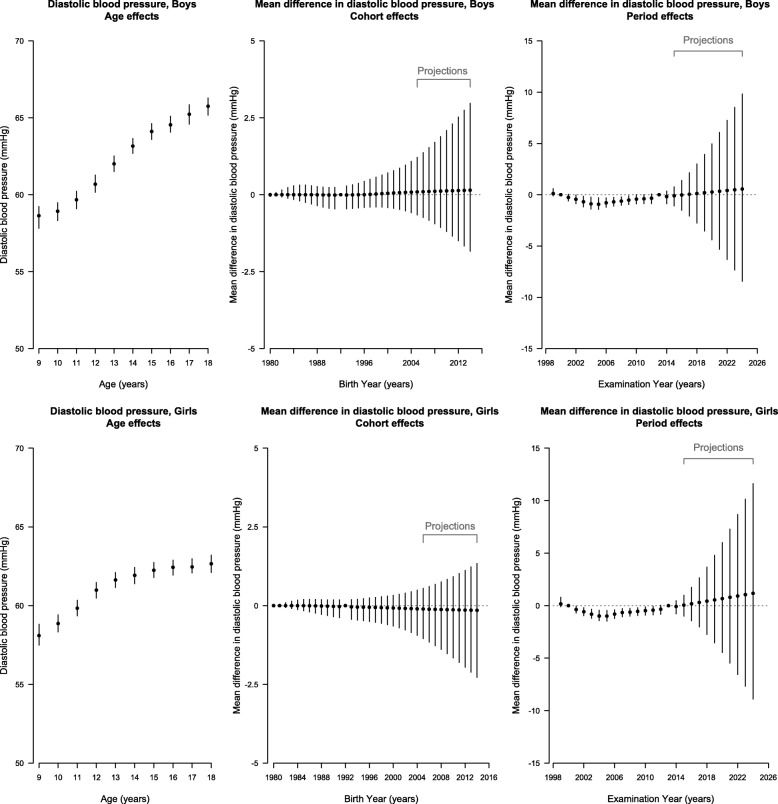
Parameter Estimates of Age, Period and Cohort Effects (Black Points) on Diastolic Blood Pressure With 95% Credible Intervals (Vertical Lines) Among Boys (Top Panel) and Girls (Bottom Panel) in Hong Kong Using Age-Period-Cohort Linear Regression with Bayesian Inference. Left (Age Effects): Estimated Diastolic Blood Pressure at Each Age From 9 to 18 Years. Middle (Cohort Effects) and Right (Period Effects): Estimated Mean Difference in Diastolic Blood Pressure for Each Birth Cohort Born From 1980–1981 to 2004–2005 (Labeled as Earliest Birth Year for Each Cohort Group) With Projected Cohort Effects for Birth Cohort Born from 2005-2006 to 2014–2015 and for Examination Periods From 1999 to 2014 With Projected Period Effects from 2015 to 2024. The Second (2000) and the Penultimate (2013) Periods and the Central Birth Cohort (1992–1993) Were Specified as the Reference Categories

Period contributed to almost all trends, apart from diastolic BP in girls, and cohort contributed in girls but not boys after accounting for age (Additional file 1: Table S1), however the full age-period cohort model was also a reasonable fit, so for consistency, a full age-period-cohort model was used for each item. Figures 2 and 3, in conjunction with the curvature plots (Additional file 1: Figure S1 and S2), show systolic and diastolic BP had a slight upward inflection at about age 9 years in girls followed by a downward inflection at about 12 years. Consistent with the later puberty in boys than girls [25], these same inflections for systolic and diastolic BP occurred about 2 years later, with an upward inflection at about age 10 years and a downward inflection at about 14 years in boys. During the period, systolic and diastolic BP had an upward inflection around 2005 in both sexes, with possibly some addition downward inflections for systolic BP in boys in about 2002 and 2012. Systolic BP appeared to have an upward inflection for boys born in about 1993 but a downward inflection for girls born in about 1998. During the projection period, systolic BP in boys was projected to decrease slightly to 2024, but diastolic BP in boys and both systolic and diastolic BP in girls were expected to continue increasing to 2024. The birth cohort effect on systolic BP was projected to continue increasing in boys but continue decreasing in girls to those born in 2014–2015, whereas the birth cohort effect on diastolic BP in both sexes mostly remained unchanged. Overall, diastolic BP was projected to continue increasing gradually to 2024 in girls and boys, whereas systolic BP increased in girls but remained stable in boys (Fig. 1).

**Fig. 4:**
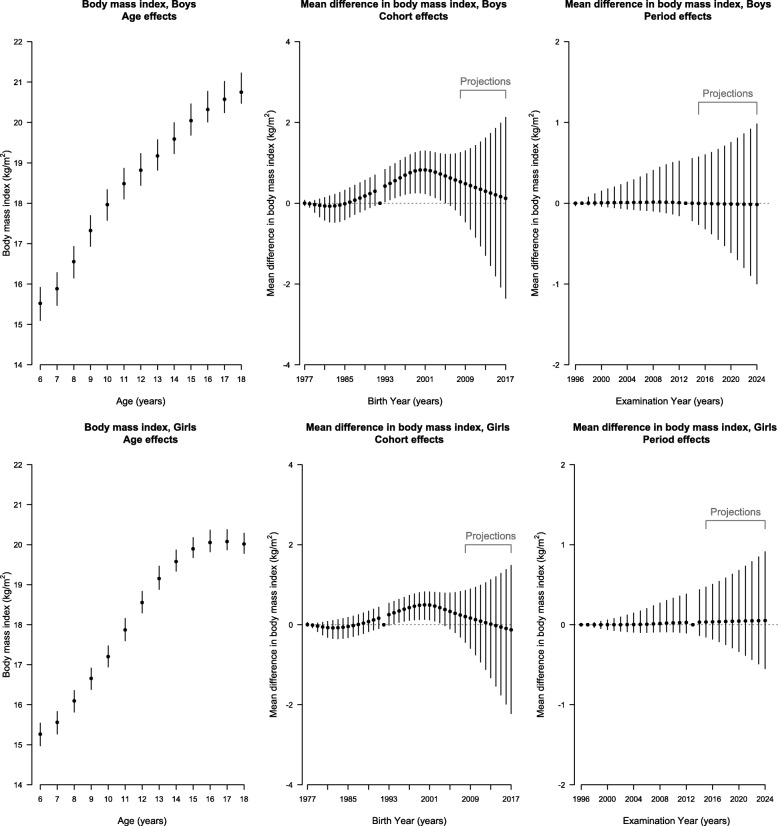
Parameter Estimates of Age, Period and Cohort Effects (Black Points) on Body Mass Index With 95% Credible Intervals (Vertical Lines) Among Boys (Top Panel) and Girls (Bottom Panel) in Hong Kong Using Age-Period-Cohort Linear Regression with Bayesian Inference. Left (Age Effects): Estimated Body Mass Index at Each Age From 6 to 18 Years. Middle (Cohort Effects) and Right (Period Effects): Estimated Mean Difference in Body Mass Index for Each Birth Cohort Born From 1977–1978 to 2007–2008 (Labeled as Earliest Birth Year for Each Cohort Group) With Projected Cohort Effects for Birth Cohort Born from 2008–2009 to 2017–2018 and for Examination Periods From 1996 to 2014 With Projected Period Effects from 2015 to 2024. The Second (1997) and the Penultimate (2013) Periods and the Central Birth Cohort (1992–1993) Were Specified as the Reference Categories

Figure 4, in conjunction with the curvature plots (Additional file 1: Figure S3), shows BMI had an upward inflection at about age 7 years in boys and girls and a downward inflection at about 11 years in boys and about 13 years in girls. There was no clear inflection point for the period effect in either sex, and accordingly the period effect was projected to remain unchanged. BMI had an upward inflection for both sexes born in about 1983 and then a downward inflection for those born in about 1998. The cohort effect for BMI in both sexes was projected to continue decreasing to those born in 2014–2015. Overall, BMI was projected to be higher in 2015 and then decrease to 2024 (Fig. 1).

In addition, the sex-specific jointpoint analyses showed that calendar years or ages when slopes of the overall trends or estimated APC effects on BP or BMI changed were similar to the APC curvature plots (Additional file 1: Table S2).

## Discussion

In Hong Kong, a currently developed Chinese setting, diastolic BP was projected to increase, but systolic BP was projected to increase only in girls, and BMI in both sexes was projected to decrease from 2015 to 2024. The projections were consistent with recent divergent trends in BP and BMI from 1996/99 to 2014. Population-wide period effects were most clearly evident for BP, whereas birth cohort-specific effects were relevant for BP and BMI.

The projected trends in BP and BMI from 2015 to 2024 represent a continuation of previous trends. It might seem counterintuitive that systolic BP in girls and diastolic BP in both sexes were projected to increase and systolic BP in boys was projected to stagnate, whereas BMI in both sexes is projected to decrease. Given BMI cannot indicate body composition and abdominal fatness, the projected decrease in BMI might not necessarily indicate a decrease in total or abdominal fat mass. However, a previous study showed fat mass may have increased more than lean mass in Chinese children in recent decades [26]. As such, our projection of decrease in BMI likely reflects decrease in fat mass and hence cannot explain the projected increase in BP.

The trends in BP and BMI from 1996/99 to 2014 showed a falling-rising trend for BP and a rising-falling trend for BMI overall. Similar discordant changes in BP and BMI over time were observed in the United States where BP fell from 1963 to 1998 before rising after a 10-year lag after BMI increased from 1963 to 2002 [2]. Several possible drivers were speculated, including a lagged effect of BMI, changing health norms within the family, early life exposures (e.g. restricted salt intake in infancy), rather than sodium intake and physical inactivity which increased in the West [2]. The exact mechanisms by which BP decreased amid rising BMI remain unknown, hence this investigation.

To explore the potential drivers of changing trends in BP and BMI in Hong Kong Chinese children and adolescents, we decomposed the trends into age, period and cohort effects. Our results indicated both population-wide and cohort-specific factors relevant to BP and cohort-specific factors relevant to BMI. The falling-rising trend for BP is unlikely to be due to conventional diet and lifestyle factors, given high salt intake [27], low fruit and vegetables intake [27] and physical inactivity [28] in Hong Kong are not known to have changed when (2004/5) the BP trend reversed. An inverse association of birth weight with systolic BP was observed [29], but was not found in a Mendelian randomization study, suggesting birth weight is unlikely a causal factor for BP [30]. Alternatively, air pollution may have worsened in Hong Kong, with increasing pollutants from neighboring Chinese provinces due to increasing energy use with economic development [31]. We have previously identified that some constituents of air pollution in Hong Kong have sex-specific associations with later pubertal timing [32], perhaps because of the trade-off between drivers of fertility and longevity [33]. A Mendelian randomization study showed later pubertal timing may be relevant to lower BP and BMI especially among girls [34]. Given child’s BMI may be more responsive to diet and lifestyle, air pollution might partly contribute to the intriguing trends of declining BP despite rising BMI, but the trends could be reversed when the lagged effect of BMI on BP occurs.

Conversely, the rising-falling trend for BMI may be related to cohort-specific factors. BMI increased since the 1983 birth cohort. In Hong Kong, there were great improvement of pediatric services including more neonatal intensive care units, genetic counselling and prenatal diagnosis since the 1980s [35]. All of these might contribute to faster early postnatal growth, which is associated with higher childhood BMI [36]. Afterwards, BMI decreased from the 1998 birth cohort, whether that is related to experience of the Asian financial crisis in utero seems unlikely. Alternatively, implementation of school-based health promotion programmes seems a more plausible explanation, such as “EatSmart@school.hk” launched in 2006 targeting primary schools with healthier lunch and snacks and improving students’ diet [37] given cohorts born in the late 1990s likely attended primary schools when the programme was introduced. As such, while living conditions are becoming more conducive to adiposity, changing attitudes, behavior and environments for successive cohorts who are increasingly exposed to school-based health promotion campaign could contribute to the recent decline in BMI.

Several limitations are noted. First, APC analyses are descriptive. We can only speculate about the potential factors related to the observed changes in BP and BMI. We could not empirically examine whether concomitant changes in trends of the proposed factors were the actual driving forces given the lack of detailed information. Nonetheless, the ecological findings are particularly helpful in hypothesis generating considering the largely unexplained divergent trends of BP and BMI. Second, APC models provide exploratory insights into the relative contribution of early life and contemporaneous exposures; yet interaction between early and later exposures or their effects acting cumulatively over life course is possible. Thirdly, given the well-known non-identifiability problem, several APC models have been developed with different assumptions, constraints and identification strategies including partial least square, mixed-effect modeling or hierarchical modeling to estimate and interpret the overall trends (first-order effect), while the Holford method estimates first-order effect but interprets only changes in trends (second-order effects). Findings may differ between methods focusing on first-order or second-order effects. To date, the Holford method with Bayesian inference has been widely used to describe trends and generate projections of disease incidence and mortality [18, 38], because of concerns that the non-unique solution to an APC model can be rotated to generate directionally different trends. We provided detailed information about the APC linear regression with Bayesian inferences used in this study to facilitate evaluation of model specification and result credibility, and, perhaps conservatively, interpreted the turning points not the trends. Finally, the projections were made by assuming future trends would depend on very recent trends; however political, economic and social conditions could change dramatically and unexpectedly, such as in response to the protests in Hong Kong in 2014 and 2019, or in response to new initiatives for population health promotion, such as Hong Kong’s Action on Salt and Sugars Reduction [39].

## Conclusions

In this recently developed Chinese setting, BP in children and adolescents was projected to increase apart from systolic BP in boys, whereas BMI was projected to decrease. Changes in contemporaneous population-wide factors may be relevant to BP, while changes in cohort-specific factors may be relevant to systolic BP and BMI. Considering drivers of BP and BMI are multifactorial and the prevalence of such factors are changing over time, the biological mechanisms underlying the divergent trends of BP and BMI remain to be elucidated.

## Supplementary information

**Additional file 1.** The age-period-cohort (APC) model

## Data Availability

The data that support the findings of this study are provided by the Student Health Service of the Department of Health, Government of the Hong Kong SAR but restrictions apply to the availability of these data, which were used under license for the current study, and so are not publicly available.

## Abbreviations

APC: Age-period-cohort
BMI: Body mass index
BP: Blood pressure
DIC: Deviance information criterion
SAR: Special Administrative Region
