# Supplementary material for: Age-period-cohort projection of trends in blood pressure and body mass index in children and adolescents in Hong Kong
Source: BMC Pediatr. 2020 Jan 29;20:43. doi: 10.1186/s12887-020-1928-2 (PMC6990538; doi:10.1186/s12887-020-1928-2)
Supplement: Supplementary file 1 — Additional file 1. The age-period-cohort (APC) model [file 12887_2020_1928_MOESM1_ESM.docx]

Appendix 1.

**The age-period-cohort (APC) model**

We decomposed the temporal trends in systolic or diastolic blood pressure (BP) for boys or girls aged from 9 to 18 (10 ages) in examination years from 1999 to 2014 (16 periods) who were born from 1980-1981 to 2004-2005 (25 birth cohorts), and body mass index (BMI) for boys or girls aged from 6 to 18 (13 ages) in examination years from 1996 to 2014 (19 periods) who were born from 1977-1978 to 2007-2008 (31 birth cohorts) in Hong Kong into three time elements, namely chronological age (age effect), examination period (period effect) and birth cohort (cohort effect) using the age-period-cohort (APC) linear regression model with Bayesian inference.

The outcomes were aggregated by cross-tabulation of age and period, in which diagonals indicated cohorts. We used mean systolic and diastolic BP aggregated for 160 combinations resulted form 10 ages, 16 periods and 25 cohorts. We used mean BMI aggregated for 247 combinations resulted form 13 ages, 19 periods and 31 cohorts.

*Model specification*

We assumed that systolic or diastolic BP or BMI for boys or girls at age *i* in examination period *j* from birth cohort *k*, followed a Gaussian distribution with mean μij and precision τij which is a reciprocal of variance, i.e., $\frac{1}{\sigma2}$ where σ^2^ was given a uniform (0, 10) prior. Under the full age-period-cohort model, the mean is specified as:

μij = αagei + βperiodj + γcohortk

where αagei (*i* = 1, ... , *I*, *I* = 10 for BP and *I* = 13 for BMI) is the age effect, βperiodj is the period effect (*j* = 1, ... , *J*, *J* = 16 for BP and *J* = 19 for BMI), and γcohortk is the cohort effect (*k* = 1, ... , *K*, *K* = 25 for BP and *K* = 31 for BMI). Including projections for *N*=10 further periods from 2015 to 2024, the fitted and projected BP or BMI were obtained through a recombination of the smoothed age αagei, period βperiodj and cohort γcohortk effects based on the relationship:

μij = αagei + βperiodj + γcohortk 1 < *i* < *I* x (*J* + *N*), *N*=10

*Autoregressive priors for age, period and cohort effects*

Under the Bayesian framework, we specified the Gaussian autoregressive priors with truncated distribution for the I age effects ai,

α1 ~ normal (0, σ2α), 0 < α1 < *∞*

α2 | α1 ~ normal (0, σ2α), 0 < α2 < *∞*

α*i* | α1, ... , α*i* -1 ~ normal (2α*i* -1 - α*i* -2, σ2α), 0 < α*i* < *∞*; 3 < *i* < *I*

where the hyperparameter$\frac{1}{\sigma2\alpha}$ is a precision parameter controlling the degree of smoothing on each time scale and was given a non-informative uniform (0, 10) prior for BP and non-informative uniform (0, 5) prior for BMI.

We used similar prior distributions without truncation for the period effects β*_j_* and the cohort effects γ*_k_* with the

hyperparameters $\frac{1}{\sigma2\beta}$ and $\frac{1}{\sigma2\gamma}$ , respectively. The autoregressive priors provided nonparametric smoothing of the estimated age, period and cohort effects, and allowed extrapolation of future period and cohort effects based on the most recent 2 period and cohort effects, respectively.

*Parameter estimation*

We obtained all parameter estimates by Markov chain Monte Carlo (MCMC) simulation methods. In the simulations, 5 chains each with 300,000 iterations were run with the first 100,000 iterations of each chain used as a burn-in period to minimize the effect of initial values for BP and 5 chains each with 500,000 iterations were run with the first 200,000 iterations of each chain used as a burn-in period for BMI. Initial values for each chain were randomly chosen from truncated normal (0,10) distributions for αagei and normal (0,10) distributions for βperiodj and γcohortkfor both BP and BMI, with uniform (0,10) distribution for σ_α_, σ_β_ and σ_γ_ for BP and uniform (0,5) distribution for σ_α_, σ_β_ and σ_γ_ for BMI. We monitored the convergence by comparing the posterior distributions across the multiple chains (which had started at different values) with convergence defined as the point when the statistic Rhat fell below the threshold of 1.2. We sampled every 10th value (‘thinning’) after the burn-in period rather than every single value to reduce the autocorrelation in the sampled values and also to reduce memory storage requirements, and a total of 200,000 sample values for BP and 300,00 sample values for BMI were retained from the posterior distributions and used for parameter inference. All fitted age, period and cohort effects and projected systolic or diastolic BP or BMI were summarized by the posterior means, and 95% credible intervals were calculated from the 2.5th percentile and 97.5th percentile of the sampled values.

Appendix Table 1. Deviance Information Criterion (DIC) Values^a^ of Different Combinations for Age, Period and Cohort Models for Systolic and Diastolic Blood Pressure (BP) Among Boys and Girls Aged 9 to 18 From 1999 to 2014 and Body Mass Index Among Boys and Girls Aged 6 to 18 From 1996 to 2014 in Hong Kong

| Outcome | Model | Components | DIC |
| --- | --- | --- | --- |
| **Systolic BP** | Girls |  |  |
|  | 1 | Age | 976.6 |
|  | 2 | Period | 981.6 |
|  | 3 | Cohort | 978.6 |
|  | 4 | Age-period | 976.0 |
|  | 5 | Age-cohort | 977.4 |
|  | 6 | Age-period-cohort (Full model) | **975.8** |
|  | Boys |  |  |
|  | 1 | Age | 1015.5 |
|  | 2 | Period | 1016.0 |
|  | 3 | Cohort | 1016.4 |
|  | 4 | Age-period | **1014.6** |
|  | 5 | Age-cohort | 1014.7 |
|  | 6 | Age-period-cohort (Full model) | 1015.6 |
| **Diastolic BP** | Girls |  |  |
|  | 1 | Age | 1065.4 |
|  | 2 | Period | 1060.6 |
|  | 3 | Cohort | 1054.7 |
|  | 4 | Age-period | 1055.5 |
|  | 5 | Age-cohort | **1052.0** |
|  | 6 | Age-period-cohort (Full model) | 1057.0 |
|  | Boys |  |  |
|  | 1 | Age | 1026.3 |
|  | 2 | Period | **1020.9** |
|  | 3 | Cohort | 1030.3 |
|  | 4 | Age-period | 1026.2 |
|  | 5 | Age-cohort | 1025.6 |
|  | 6 | Age-period-cohort (Full model) | 1024.5 |
| **BMI** | Girls |  |  |
|  | 1 | Age | 1766.9 |
|  | 2 | Period | 1769.8 |
|  | 3 | Cohort | 1767.5 |
|  | 4 | Age-period | 1755.0 |
|  | 5 | Age-cohort | 1778.7 |
|  | 6 | Age-period-cohort (Full model) | **1750.1** |
|  | Boys |  |  |
|  | 1 | Age | 1728.1 |
|  | 2 | Period | 1730.2 |
|  | 3 | Cohort | 1741.6 |
|  | 4 | Age-period | **1720.9** |
|  | 5 | Age-cohort | 1752.9 |
|  | 6 | Age-period-cohort (Full model) | 1728.6 |

^a^DIC was obtained based on different combinations of age-period-cohort models using Markov chain Monte Carlo (MCMC) simulation methods with 5 concurrent chains, each with 300,000 iterations (the first 100,000 iterations as a burn-in period) for BP, and each with 500,000 iterations (the first 200,000 iterations as a burn-in period) for BMI

Appendix Table 2. Number of Jointpoints Identified for Overall Trends and Parameter Estimates of Age, Period and Cohort Effects of Systolic and Diastolic Blood Pressure (BP) Among Boys and Girls aged 9 to 18 from 1999 to 2014 and Body Mass Index (BMI) Boys and Girls aged 6 to 18 from 1996 to 2014 With Projected BP and BMI to 2024 in Hong Kong using Jointpoint Analyses with Modified Bayesian Information Criterion^a^

|  |  | Systolic BP | | Diastolic BP | | BMI | | | |
| --- | --- | --- | --- | --- | --- | --- | --- | --- | --- |
|  |  | Girls | Boys | Girls | Boys | Girls | | Boys | |
| **Overall trends** | Number of jointpoints | 1 | 2 | 2 | 1 | 5 | 5 | |  |
|  | Years of jointpoints | **2005** | **2005, 2012** | **2000, 2004** | **2003** | **1997, 1999, 2011, 2014, 2015** | **1997, 1999, 2011, 2014, 2015** | |  |
|  | Segmented periods | 1999-2005 | 1999-2005 | 1999-2000 | 1999-2003 | 1996-1997 | 1996-1997 | |  |
|  |  | 2005-2024 | 2005-2012 | 2000-2004 | 2003-2024 | 1997-1999 | 1997-1999 | |  |
|  |  |  | 2012-2024 | 2004-2024 |  | 1999-2011 | 1999-2011 | |  |
|  |  |  |  |  |  | 2011-2014 | 2011-2014 | |  |
|  |  |  |  |  |  | 2014-2015 | 2014-2015 | |  |
|  |  |  |  |  |  | 2015-2024 | 2015-2024 | |  |
| **Age effect** | Number of jointpoints | 1 | 2 | 1 | 0 | 1 | 1 | |  |
|  | Years of jointpoints | **13** | **10, 14** | **13** | **NA** | **15** | **11** | |  |
|  | Segmented ages | 9-13 | 9-10 | 9-13 | NA | 6-15 | 6-11 | |  |
|  |  | 13-18 | 10-14 | 13-18 |  | 15-18 | 11-18 | |  |
|  |  |  | 14-18 |  |  |  |  | |  |
| **Period effect** | Number of jointpoints | 1 | 1 | 1 | 1 | 0 | 2 | |  |
|  | Years of jointpoints | **2005** | **2002, 2005, 2012** | **2004** | **2004** | **NA** | **2010, 2013** | |  |
|  | Segmented periods | 1999-2005 | 1999-2002 | 1999-2004 | 1999-2004 | **NA** | 1996-2010 | |  |
|  |  | 2005-2024 | 2002-2005 | 2004-2024 | 2004-2024 |  | 2010-2013 | |  |
|  |  |  | 2005-2012 |  |  |  | 2013-2024 | |  |
|  |  |  | 2012-2024 |  |  |  |  | |  |
| **Cohort effect** | Number of jointpoints | 3 | 1 | 1 | 1 | 2 | 3 | |  |
|  | Years of jointpoints | **1989, 1996, 2006** | **1992, 2004** | **1988** | **1995, 2003** | **1985, 2000** | **1985, 2001** | |  |
|  | Segmented birth years | 1980-1989 | 1980-1992 | 1980-1988 | 1980-1995 | 1977-1985 | 1977-1985 | |  |
|  |  | 1989-1996 | 1992-2004 | 1988-2014 | 1995-2003 | 1985-2000 | | 1985-2001 | |
|  |  | 1996-2006 | 2004-2014 |  | 2003-2014 | 2000-2017 | | 2001-2017 | |
|  |  | 2006-2014 |  |  |  |  | |  | |

Abbreviations: BP: blood pressure, BMI: body mass index, CI, confidence interval, NA, not applicable

^a^ Modified Bayesian Information Criterion (BIC) identifies the fitted model by penalizing extra parameters in model to avoid overfitting and can handle irregularities of likelihood function; lower modified BIC indicates better fitted model, with BIC decreases with reduced unexplained variation in dependent variable and fewer explanatory variables. The fitted model would be the parsimonious model with minimally sufficient number of jointpoints.

Appendix Figure 1. Curvature Components for Age, Period and Cohort Effects (Black Points) on Systolic Blood Pressure With 95% Credible Intervals (Vertical Lines) Among Boys (Top Panel) and Girls (Bottom Panel) in Hong Kong Using Age-Period-Cohort Linear Regression with Bayesian Inference. Left (Age Effects): Curvature at Each Age From 9 to 18 Years. Middle (Cohort Effects) and Right (Period Effects): Curvature for Each Birth Cohort Born From 1980-1981 to 2004-2005 (Labeled as Earliest Birth Year for Each Cohort Group) With Projected Curvature for Birth Cohort Born from 2005-2006 to 2014-2015 and for Examination Periods From 1999 to 2014 With Projected Curvature from 2015 to 2024.

Appendix Figure 2. Curvature Components for Age, Period and Cohort Effects (Black Points) on Diastolic Blood Pressure With 95% Credible Intervals (Vertical Lines) Among Boys (Top Panel) and Girls (Bottom Panel) in Hong Kong Using Age-Period-Cohort Linear Regression with Bayesian Inference. Left (Age Effects): Curvature at Each Age From 9 to 18 Years. Middle (Cohort Effects) and Right (Period Effects): Curvature for Each Birth Cohort Born From 1980-1981 to 2004-2005 (Labeled as Earliest Birth Year for Each Cohort Group) With Projected Curvature for Birth Cohort Born from 2005-2006 to 2014-2015 and for Examination Periods From 1999 to 2014 With Projected Curvature from 2015 to 2024.

Appendix Figure 3. Curvature Components of Age, Period and Cohort Effects (Black Points) on Body Mass Index With 95% Credible Intervals (Vertical Lines) Among Boys (Top Panel) and Girls (Bottom Panel) in Hong Kong Using Age-Period-Cohort Linear Regression with Bayesian Inference. Left (Age Effects): Curvature at Each Age From 6 to 18 Years. Middle (Cohort Effects) and Right (Period Effects): Curvature for Each Birth Cohort Born From 1977-1978 to 2007-2008 (Labeled as Earliest Birth Year for Each Cohort Group) With Projected Curvature for Birth Cohort Born from 2008-2009 to 2017-2018 and for Examination Periods From 1996 to 2014 With Projected Curvature from 2015 to 2024.
